# Burden of respiratory syncytial virus in spinal muscular atrophy: a retrospective nationwide cross-sectional and birth cohort study

**DOI:** 10.1186/s13023-026-04494-0

**Published:** 2026-07-14

**Authors:** Hao-Wei Chung, Chen-Hua Wang, Chia‑Hung Yu, Chiao-Yun Huang, Fu-Wen Liang

**Affiliations:** 1 Department of Pediatrics, Kaohsiung Medical University Hospital, Kaohsiung Medical University, Kaohsiung, Taiwan; 2 Department of Pediatrics, School of Medicine, College of Medicine, Kaohsiung Medical University, Kaohsiung, Taiwan; 3 Department of Biological Science and Technology, National Yang Ming Chiao Tung University, Hsinchu, Taiwan; 4 Department of Pediatrics, Kaohsiung Municipal Siaogang Hospital, Kaohsiung Medical University, Kaohsiung, Taiwan; 5 Department of Anesthesiology, Chi Mei Medical Center, Tainan, Taiwan; 6 Department of Computer Science and Information Engineering, Southern Taiwan University of Science and Technology, Tainan, Taiwan; 7 Department of Public Health, College of Health Sciences, Kaohsiung Medical University, No.100, Shih- Chuan 1st Road, Sanmin Dist, Kaohsiung, 807378 Taiwan; 8 Department of Medical Research, Kaohsiung Medical University Hospital, Kaohsiung Medical University, Kaohsiung, Taiwan; 9 Center for Big Data Research, Kaohsiung Medical University, Kaohsiung, Taiwan

**Keywords:** Respiratory syncytial virus, Spinal muscular atrophy, Hospitalization

## Abstract

**Background:**

Spinal muscular atrophy (SMA) is a severe neuromuscular disease associated with a high risk of respiratory complications, yet real-world data on respiratory syncytial virus (RSV) infection in this population remain limited. This study aimed to investigate the severity, hospitalization costs, and seasonal patterns of RSV infections in patients with SMA using real-world data in Taiwan.

**Methods:**

This retrospective study analyzed data from Taiwan’s National Health Insurance Research Database from 2009 to 2021. Patients with SMA were identified through at least two outpatient clinic visits and one hospital admission. The analysis included a cross-sectional study and a birth cohort analysis for sensitivity testing. RSV-associated hospitalizations were identified using diagnostic codes for RSV. The main outcomes included ICU admissions, ventilator use, hospitalization costs, timing of RSV onset, and seasonal infection trends.

**Results:**

This study documented 914 hospitalizations in patients with SMA over 13 years, including 14 RSV infections (1.5%). All RSV-related hospitalizations were documented before age 18. In a birth cohort analysis comparing 110 patients with SMA to 2,103,997 individuals from the general population, patients with SMA demonstrated significantly higher RSV hospitalization rates (9.8% vs. 1.6%), ICU admissions (54.5% vs. 4.7%; *p* < 0.0001), and ventilator use (54.5% vs. 1.7%; *p* < 0.0001), with a later mean onset (2.54 ± 2.05 years vs. 1.05 ± 1.05 years; *p* = 0.0073) and substantially higher hospitalization costs (median 967 [560-6,462] USD vs. 683 [487–1002] USD; *p* = 0.0480). Infections peaked in fall and winter (72.7%) for patients with SMA. ICU admission rates for RSV increased with age and decreased with later SMA onset.

**Conclusions:**

Patients with SMA are at heightened risk for severe RSV infections even beyond infancy, underscoring the need for age-specific and seasonal prevention strategies, particularly for those over two years old.

**Supplementary Information:**

The online version contains supplementary material available at https://doi.org/10.1186/s13023-026-04494-0.

## Background

Among neuromuscular diseases (NMD), spinal muscular atrophy (SMA) is one of the most common and clinically significant conditions, not only due to its historically severe and often fatal natural history, but also because of the groundbreaking advancements in its treatment [1]. SMA is an autosomal recessive neurodegenerative disease caused by mutations in the SMN1 gene, resulting in the loss of motor neurons and leading to progressive muscle wasting and weakness [2, 3]. The phenotype has historically been classified into five grades of severity (SMA type 0 through type 4) based on age of onset and the best motor function achieved in the natural history without treatment. Type 1 is the most common severe form with onset prior to 6 months of age, accounting for 60% of patients with SMA; without treatment, death frequently occurs before 2 years of age due to acute respiratory failure or pneumonia. SMA was once the leading genetic cause of infant death worldwide, but with the advent of disease-modifying treatments, the clinical trajectory and motor outcomes of patients with SMA have significantly improved [1, 4]. The worldwide incidence of SMA is approximately 7.8 to 10 cases per 100,000 live births [5]. The prevalence of SMA was estimated to be 0.85 per 100,000 persons aged 0–79 years in Canada [6].

Respiratory complications are the leading cause of morbidity and mortality in SMA [7]. SMA-induced muscle weakness predominantly affects expiratory muscles while relatively sparing inspiratory muscles, resulting in ineffective cough and impaired airway clearance [7]. This imbalance leads to mucus retention, recurrent atelectasis, and an increased susceptibility to lower respiratory tract infections (LRTIs). Effective airway clearance strategies, including cough-assist devices and chest physiotherapy, are essential components of respiratory management in this population. Concomitant restrictive lung disease, thoracic deformities, and bulbar dysfunction further exacerbate respiratory vulnerability through compromised ventilation mechanics and microaspiration [7, 8]. Unlike many other neuromuscular disorders, this distinctive respiratory pathophysiology places patients with SMA at heightened risk of early and recurrent LRTIs, providing additional justification for investigating RSV burden specifically in the SMA population rather than grouping SMA within the broader NMD category [7]. Given the high burden of respiratory infections in SMA, preventive strategies including immunization and infection control are considered essential components of standard care [9].

Respiratory syncytial virus (RSV), a common seasonal respiratory virus, is the leading cause of severe LRTI in young children and older adults worldwide [10] In 2019 alone, RSV caused an estimated 33.1 million LRTI episodes, 3.6 million hospital admissions, and 26,300 in-hospital deaths in children under 5 years of age worldwide, with a disproportionate burden on low- and middle-income countries [10]. Infants under 12 months and children with conditions such as prematurity, congenital heart disease, or genetic disorders are particularly vulnerable to severe RSV infection [11–14]. Despite clinical guidelines in high-income countries recommend RSV immunoprophylaxis for high-risk groups including patients with SMA, real-world data describing the burden and severity of RSV infection specifically in patients with SMA remains limited [9]. Previous studies have examined SMA within the broader NMD category, limiting understanding of the unique clinical characteristics and outcomes of RSV infection in this population [11].

In Taiwan, disease-modifying treatments (DMT) for SMA became available only recently. Nusinersen (Spinraza) was the first DMT approved for SMA, with National Health Insurance (NHI) reimbursement initiated in July 2020 for patients diagnosed before 12 months of age and treated at ≤ 6 years of age. Risdiplam (Evrysdi) and onasemnogene abeparvovec (Zolgensma) received NHI coverage in 2021 and 2023, respectively. Considering the recent breakthroughs in SMA treatment that have significantly improved the cwith SMA [15], it is essential for individuals with SMA and their families to proactively plan strategies to prevent RSV-related emergencies. To address this gap, we utilized Taiwan’s nationwide health insurance database to investigate the severity, hospitalization costs, and seasonal patterns of RSV infections specifically in patients with SMA over 13 years, comparing outcomes with those of the general population and other lower respiratory tract infections.

## Methods

### Study design and study population

This retrospective nationwide study utilized data from the National Health Insurance Research Database (NHIRD) in Taiwan between 2009 and 2021 [16]. This observation window was selected to maintain consistent data availability for analysis. This period includes both ICD-9-CM (2009–2015) and ICD-10-CM (2016–2021) coding systems to maximize case identification and largely reflects the pre–DMT era for SMA in Taiwan. All patients with SMA were confirmed by at least two outpatient clinic visits and one hospital admission with SMA diagnosis codes (ICD-10-CM: G12.0, G12.1, G12.9). The NHIRD covers medical records for approximately 99% of Taiwan’s population (23.74 million residents), with an estimated capture rate of 97%. [16] First, we used a cross-sectional approach by extracting hospital healthcare records from 2009 to 2021 to calculate the rate of RSV-associated hospitalizations in all patients with SMA under the age of 70. Second, to identify the possible age range of SMA diagnosis, we conducted a sensitivity analysis using the birth cohort design, including only live births from 2009 to 2020, with SMA cases identified based on the same diagnostic criteria mentioned above and followed through 2021. The age of the first SMA diagnosis was categorized in the same way as the infection period. We excluded children with multiple gestations (e.g., twins, triplets), as they carry a higher baseline risk of prematurity and low birthweight, which are independent risk factors for severe RSV infection [17]; those with missing data; those who died before age 2 without RSV infections, to avoid survivorship bias; and those who had received palivizumab prophylaxis, to ensure the study population reflected natural RSV infection outcomes without the confounding effect of immunoprophylaxis.

During our study period (2009–2021), disease-modifying treatments (DMT) for SMA were largely unavailable in Taiwan. Nusinersen received limited NHI reimbursement beginning in 2020, restricted to patients with SMA type 1 diagnosed before age 2, and risdiplam and onasemnogene abeparvovec received NHI coverage in subsequent years with similarly restrictive criteria. As DMT-specific medication codes were not included in our original variable extraction, patients who may have received DMT during the final year of the study period were not specifically identified or excluded. Our findings therefore primarily reflect the burden of RSV infection in patients with SMA during the pre-treatment era. Additionally, SMA newborn screening had not been implemented as a national program in Taiwan during the study period, and the NHIRD does not contain specific indicators to distinguish patients identified through newborn screening from those diagnosed by clinical presentation.

### Variables

The outcomes, as identified in the claims data using the International Classification of Disease, Ninth Revision, Clinical Modification (ICD-9-CM) and ICD-10-CM, have been validated for accuracy in NHIRD through prior studies [18]. We analyzed healthcare records from the NHIRD between 2009 and 2021 to identify RSV-associated hospitalizations among patients with SMA. RSV infection was defined by diagnostic codes for RSV-associated bronchiolitis and viral pneumonia (E-Table 1 in the Supplement). The age at RSV infection was categorized as less than 1 year, 1 to 3 years, and 4 years or older [11]. Additionally, to provide a broader perspective on the respiratory infection burden in patients with SMA and to account for possible misclassification of RSV as unspecified viral infections due to diagnostic limitations, we included hospitalizations for viral lower respiratory tract infections (LRTI) with non-RSV or unspecified viral pathogens as a comparator group in our analysis. Mechanical ventilator use includes both invasive mechanical ventilation and non-invasive ventilation initiated or escalated during hospitalization. Hospitalization costs were calculated based on total NHI-reimbursed expenditures for each hospitalization episode. In the NHIRD, costs are recorded as reimbursement points that approximate monetary value and were used as cost measures, with values converted to United States Dollars (USD) for analysis. Patients with SMA are eligible for catastrophic illness certification under the Taiwan’s National Health Insurance Program, ensuring comprehensive coverage of medical expenses and enabling complete capture of healthcare utilization through claims data.

### Statistical analysis

The characteristics of RSV/LRTI-infected patients with SMA were compared with those of non-infected patients with SMA, as well as with the general population, using the χ² test. Additionally, the χ² test or Fisher exact test and Wilcoxon rank-sum test were used to examine differences in healthcare utilization, including infection rates, age at infection, intensive care unit (ICU) admission rates, mechanical ventilator use in ICU, length of hospital stay, and medical costs, between RSV and LRTI cases among patients with SMA. A two-sided p-value < 0.05 was considered statistically significant. We used SAS 9.4 (SAS Institute Inc., Cary, North Carolina) for data analysis.

## Results

During the study period, 914 hospitalized individuals with SMA across all age groups (0–70 years) were identified between 2009 and 2021 (E-Table 2 in the Supplement). This cross-sectional cohort included both pediatric and adult patients, reflecting the overall healthcare utilization profile of the SMA population. Among these, 20.0% had a history of mechanical ventilation and 5.9% had undergone spinal fusion surgery, serving as proxies of disease severity.

In the cross-sectional analysis of hospitalization episodes among patients with SMA **(**Table 1**)**, 14 patients (1.5%) experienced RSV infections, while 46 patients (5.0%) had LRTI (*P* < 0.0001). The mean age of RSV infection was significantly lower than that of LRTI (2.57 ± 3.03 vs. 12.20 ± 13.70 years, *p* = 0.0153). Importantly, all RSV-related hospitalizations occurred exclusively in patients under 18 years of age, whereas LRTI hospitalizations spanned both pediatric and adult age groups. Although not statistically significant, RSV infections showed higher ICU admission rates (42.9% vs. 23.9%, *p* = 0.1900) and ventilator support rates (42.9% vs. 28.3%, *p* = 0.3381) compared to LRTI cases. The mean duration of hospitalization was shorter for RSV cases (2.57 ± 3.03 days vs. 7.45 ± 7.51 days, *p* = 0.3495), whereas hospitalization costs were higher for RSV cases (median 930 [560-3,597] USD vs. 643 [376-1,204] USD, *p* = 0.0934).

**Table 1:** Comparison of RSV and LRTI infection hospitalizations among a cross-section of patients with SMA

Outcomes	RSV	LRTI	*P*-value
**All SMA N = 914)**			
Incidence, n (%)	14 (1.5)	46 (5.0)	< 0.0001
< 18 years, n (%)	14 (100.0)	34 (73.9)	
Patient age at hospitalization, years Mean ± SD (Median, Q1-Q3),	2.57 ± 3.03 (1.5, 0–4)	12.20 ± 13.70 (7.5, 1–17)	0.0153
ICU rate, n (%)	6 (42.9)	11 (23.9)	0.1900
Ventilator support rate, n (%)	6 (42.9)	13 (28.3)	0.3381
Hospital length of stay, days Mean ± SD (Median, Q1-Q3),	11.5 ± 15.0 (6.5, 4–10)	7.45 ± 7.51 (5.5, 3–8)	0.3495
Hospitalization cost, USD Mean ± SD (Median, Q1-Q3)	4,301 ± 6,804 (930, 560-3,597)	1,774 ± 3,902 (643, 376-1,204)	0.0934

Abbreviations: ICU: Intensive Care Unit; LRTI: Lower Respiratory Tract Infection; USD: United States Dollar; RSV: Respiratory Syncytial Virus; SMA: Spinal Muscular Atrophy

In the birth cohort analysis, 110 patients with SMA born between 2009 and 2020 were followed through 2021 (E-figure 1 in the Supplement). Neonatal characteristics of the SMA birth cohort **(**Table 2**)** demonstrated increased baseline respiratory vulnerability, including higher rates of low birthweight (15.2% vs. 8.9%, *p* = 0.0363), congenital anomalies (2.7% vs. 0.4%, *p* = 0.0085), NICU admission (24.6% vs. 17.1%, *p* = 0.0376), and respiratory support at birth including CPAP (7.3% vs. 3.1%, *p* = 0.0207), supplemental oxygen (10.0% vs. 4.4%, *p* = 0.0090), and mechanical ventilation (5.5% vs. 0.8%, *p* = 0.0003).

**Table 2:** Characteristics for patients with SMA and the general population in the birth cohort

Variables	SMA *N* = 110	General population *N* = 2,103,997	*P*-value
	*N* (%)	*N* (%)	
Male	52 (47.3)	1,090,628 (51.8)	0.3382
Primipara	68 (61.6)	1,316,097 (62.6)	0.8736
Singleton	110 (98.2)	2,034,176 (96.7)	0.8527
Maternal smoking	0 (0.0)	2,028 (0.1)	1.0000
Preterm	12 (11.6)	187,601 (8.9)	0.4634
Low birthweight	16 (15.2)	186,637 (8.9)	0.0363
Congenital anomalies	3 (2.7)	7,909 (0.4)	0.0085
NICU*	27 (24.6)	359,443 (17.1)	0.0376
CPAP*	8 (7.3)	64,748 (3.1)	0.0207
Oxygenation supply*	11 (10.0)	92,089 (4.4)	0.0090
Mechanical ventilation*	6 (5.5)	16,923 (0.8)	0.0003

CPAP continuous positive airway pressure; NICU: neonatal intensive care unit

*The variables refer to interventions during the birth hospitalization (neonatal period)

Compared to 2,103,997 general population births, the cumulative RSV hospitalization rate was substantially higher in patients with SMA (9.8% vs. 1.6%). RSV hospitalizations in patients with SMA were associated with markedly higher ICU admission rates (54.5% vs. 4.7%, *p* < 0.0001) and mechanical ventilator use (54.5% vs. 1.7%, *p* < 0.0001). Patients with SMA were hospitalized for RSV at an older mean age than the general population (2.54 ± 2.05 vs. 1.05 ± 1.05 years, *p* = 0.0073) and incurred higher hospitalization costs (median 967 [560-6,462] USD vs. 683 [487–1002] USD, *p* = 0.048). **(**Table 3**)**

**Table 3:** Comparison of RSV and LRTI infection hospitalizations between birth cohorts of patients with SMA and the general population

Outcomes	SMA (*N* = 110)	General population (*N* = 2,103,997)	*P*-value
**RSV hospitalization**, n (%)	11 (9.8)	34,362 (1.6)	
Patient age at hospitalization, years Mean ± SD (Median, Q1-Q3)	2.54 ± 2.05 (1.98, 0.63–4.93)	1.05 ± 1.05 (0.71, 0.32–1.39)	0.0073
ICU rate, n (%)	6 (54.5)	1,603 (4.7)	< 0.0001
Ventilator support rate, n (%)	6 (54.5)	571 (1.7)	< 0.0001
Hospital length of stay, days Mean ± SD (Median, Q1-Q3)	12.82 ± 16.76 (6.0, 4–11)	5.22 ± 2.58 (5.0, 4–6)	0.0709
Hospitalization cost, USD Mean ± SD (Median, Q1-Q3)	5,171 ± 7,491 (967, 560-6,462)	871 ± 1,101 (683, 487–1002)	0.0480
**Viral LRTI hospitalization**, n (%)	17 (15.5)	93,990 (4.5)	-
Patient age at hospitalization, years Mean ± SD (Median, Q1-Q3), years	1.84 ± 1.65 (1.23, 0.48–2.84)	1.67 ± 1.81 (1.00, 0.48–2.07)	0.3880
ICU rate, n (%)	4 (22.2)	2685 (2.9)	0.0015
Ventilator support rate, n (%)	5 (27.8)	727 (0.8)	< 0.0001
Hospital length of stay, days Mean ± SD (Median, Q1-Q3)	8.88 ± 11.53 (4.0, 3–8)	4.37 ± 2.20 (4.0, 3–5)	0.5112
Hospitalization cost, USD Mean ± SD (Median, Q1-Q3)	2,914 ± 6,230 (528, 360–980)	621 ± 631 (509, 371–717)	0.4918

Abbreviations: ICU: Intensive Care Unit; LRTI: Lower Respiratory Tract Infection; USD: United States Dollar; RSV: Respiratory Syncytial Virus; SMA: Spinal Muscular Atrophy

Similarly, viral LRTI hospitalization rates were higher in patients with SMA (16.1% vs. 4.5%), with significantly elevated ICU admission rates (22.2% vs. 2.9%, *p* = 0.0015) and ventilator support needs (27.8% vs. 0.8%, *p* < 0.0001). One death occurred in the SMA group with LRTI (5.6% mortality rate), which was not statistically significant compared to the general population (0.3%, *P* = 0.0556).

**Fig. 1:**
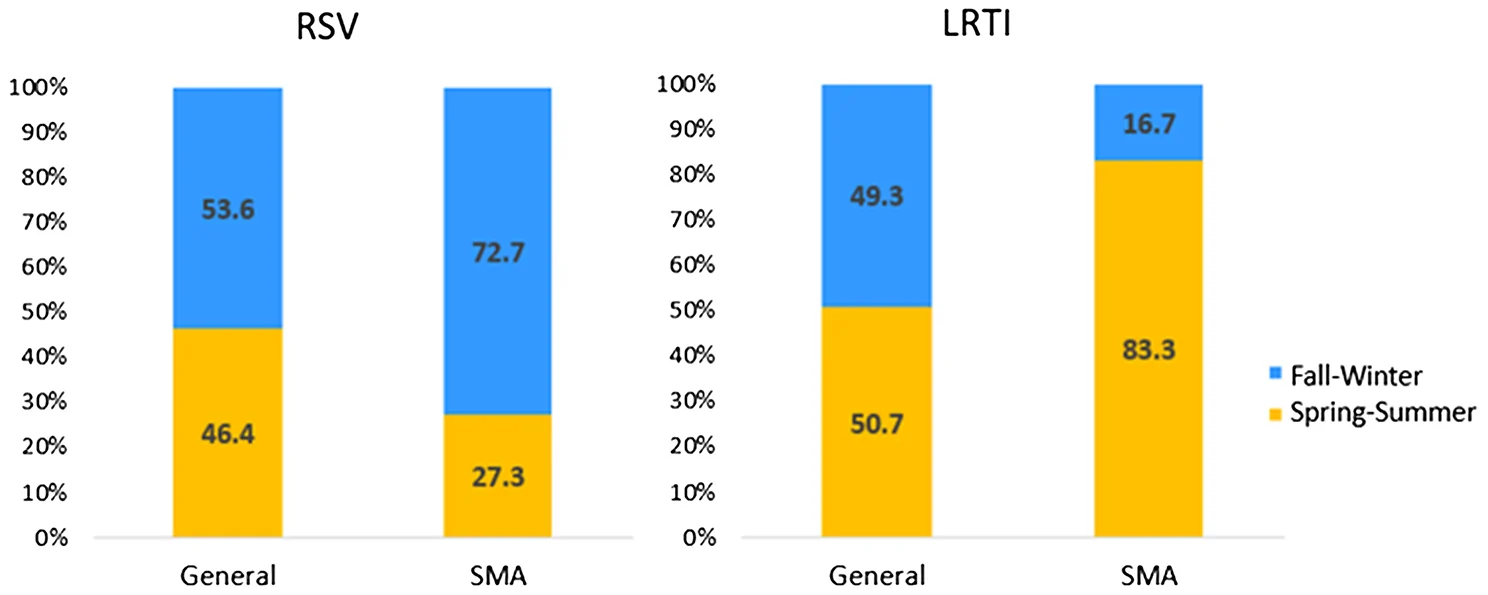
Seasonal distribution of RSV and LRTI hospitalizations in the birth cohorts of patients with SMA and the general population. Abbreviations: LRTI: Lower Respiratory Tract Infection; RSV: Respiratory Syncytial Virus; SMA: Spinal Muscular Atrophy

Seasonal patterns of respiratory infections differed between patients with SMA and the general population **(**Fig. 1**)**. For RSV, infections in the general population were distributed between fall-winter (53.6%) and spring-summer (46.4%), while patients with SMA had a higher proportion of infections during the fall-winter seasons (72.7%) compared to spring-summer (27.3%). For LRTI, the general population showed relatively even distribution between fall-winter (49.3%) and spring-summer (50.7%), whereas patients with SMA presented a distinct pattern with 83.3% of infections occurring in spring-summer and only 16.7% in fall-winter.

**Fig. 2:**
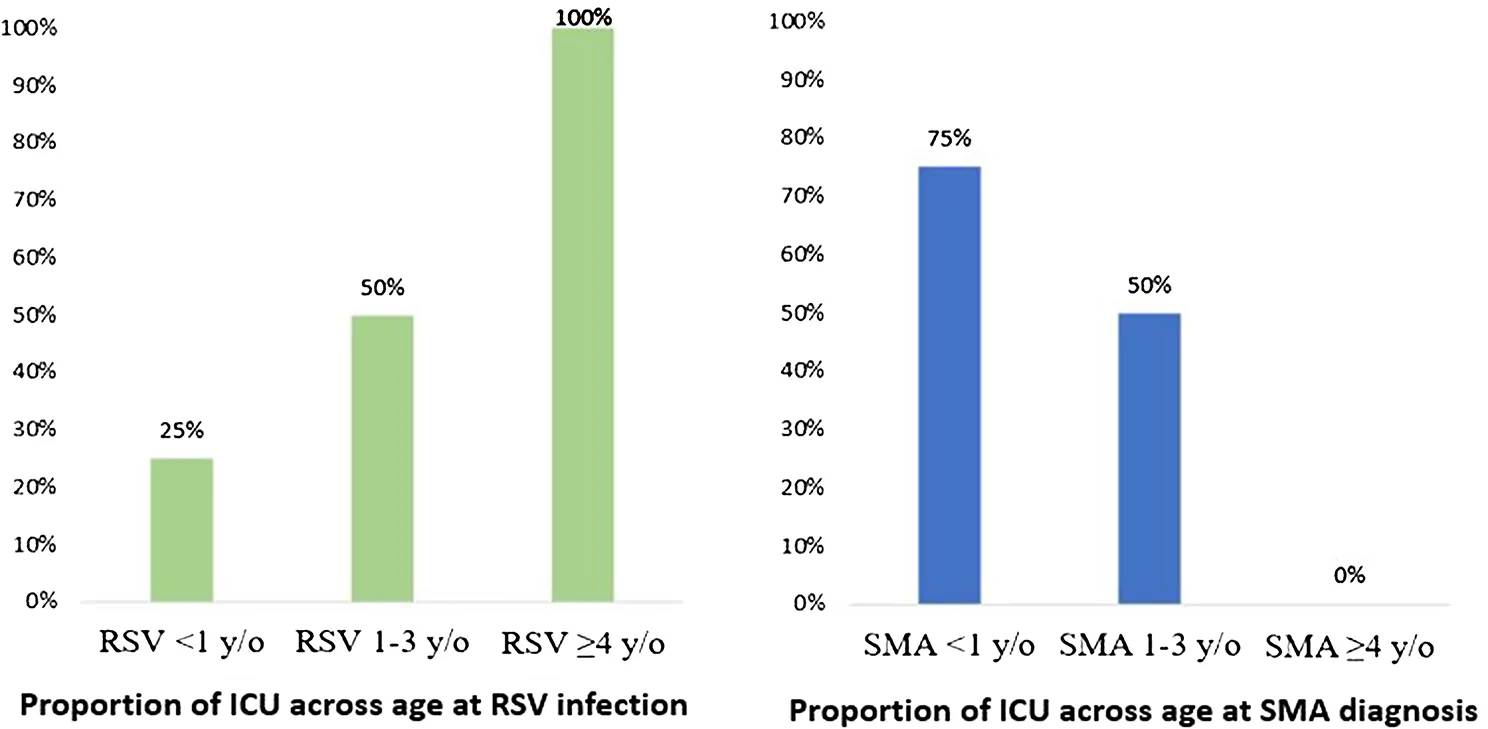
Proportion of ICU admission across age at RSV infection and at SMA diagnosis in patients with SMA. Abbreviations: ICU: Intensive Care Unit; RSV: Respiratory Syncytial Virus; SMA: Spinal Muscular Atrophy

The proportion of ICU admissions showed distinct patterns across different age groups for RSV infection and SMA onset **(**Fig. 2**)**. ICU admission rates for RSV infections showed an age-related increase, with 25% of cases in patients under 1 year, 50% in those aged 1–3 years, and 100% in patients aged 4 years or older. Conversely, ICU admissions decreased with later SMA onset, with 75% of cases in patients with SMA onset before 1 year, 50% in those aged 1–3 years, and no ICU admissions in those with SMA onset at 4 years or older.

## Discussion

In this nationwide study, RSV accounted for 1.5% of all 914 hospitalizations in patients with SMA spanning all age groups over 13 years. Since all RSV infections occurred exclusively in pediatric patients, this proportion was diluted by the inclusion of adult patients in the cross-sectional cohort. The birth cohort analysis, which provided a more age-appropriate denominator, revealed a substantially higher RSV hospitalization rate among patients with SMA (9.8%) compared to the general population (1.6%), with markedly elevated ICU admission rates and mechanical ventilation use. Distinct seasonal patterns were observed, with RSV infections in patients with SMA predominating in fall and winter. ICU admission rates for RSV increased with patient age but decreased with later SMA onset, suggesting that both age at RSV infection and age at SMA diagnosis independently contribute to the severity of RSV-related outcomes.

A meta-analysis of 17 statistical modeling studies revealed a U-shaped pattern for RSV-associated hospitalization rates across the age spectrum, with the highest burden in children under 1 year of age [19]. In contrast, children with SMA hospitalized for RSV in our study had an older mean age of 2.54 years, consistent with previous findings in children with neuromuscular disorders [20] A national cohort study in Canada found that children with neurogenic disorders exhibited a later mean age of RSV hospitalization (3.12 years) compared to those with chronic lung disease (1.18 years) [21] Despite this delayed onset, patients with SMA in our birth cohort demonstrated markedly higher rates of ICU admission (54.5% vs. 4.7%) and mechanical ventilation use (54.5% vs. 1.7%) compared to the general population, consistent with another national study reporting that 33% of children with neurologic disorders required ICU stays during RSV hospitalization [11]. Notably, ICU admission rates increased progressively across age groups, from 25% in patients under 1 year to 100% in those aged 4 years or older. This trend aligns with the progressive nature of SMA, as older children may experience declining respiratory muscle function, increasing their risk for a more severe clinical course and pulmonary compromise with RSV or other respiratory viruses [8]. 

In temperate countries, RSV seasonality typically peaks in late autumn or winter, whereas subtropical regions such as Taiwan exhibit different patterns [22]. Our data showed that RSV infections in the general population followed the expected subtropical distribution, while patients with SMA demonstrated a seasonal pattern more similar to temperate regions, with 72.7% of infections occurring in fall and winter. It is also possible that physicians may test for RSV more readily during the expected RSV season, which could amplify the apparent seasonal concentration. However, these findings should be interpreted cautiously given the small sample size and the variability of actual infection patterns, which may differ annually depending on factors such as humidity and air pollution [23]. During 2020–2021, the COVID-19 pandemic led to widespread non-pharmaceutical interventions (NPIs), including mask-wearing and social distancing, which substantially reduced RSV circulation globally. In Taiwan, a sharp decline in RSV cases was observed during this period, followed by an atypical resurgence in 2021 [22]. This temporal disruption may have affected the seasonal distribution and overall incidence of RSV infections captured in our study period, and our findings should be interpreted with this context in mind. Future research should explore whether the seasonality of RSV infection in children with SMA genuinely differs from that of the general pediatric population.

Our study provides real-world data to inform RSV prevention strategies for patients with SMA. Because DMT availability in Taiwan was extremely limited during the study period and RSV prophylaxis for SMA is not covered by the NHI, most patients in our cohort were likely untreated. In the current era of increasing DMT availability, the impact of RSV infection among DMT-treated patients warrants further investigation. Despite remarkable progress, current SMA treatments are not curative [12] and reducing the risk of severe respiratory hospitalizations remains a crucial component of comprehensive care. In a real-world setting, nirsevimab therapy was effective in reducing the risk of hospitalized RSV-associated bronchiolitis [24]. However, current monoclonal antibody prophylaxis for RSV has indications and clinical trials primarily targeting children under two years of age [24, 25]. Our findings indicate that children with SMA may experience RSV beyond two years of age, with a comparable risk of severe outcomes, consistent with observations in the broader NMD population [11, 20, 26] These results highlight the need for future studies to evaluate the protective efficacy of RSV monoclonal antibody therapies in high-risk populations older than two years, particularly among children with SMA.

The main strength of this study is the use of a nationwide database encompassing all patients with SMA who had been hospitalized over a 13-year period. Although clinical guidelines recommend RSV prophylaxis for patients with SMA, [20] it is not currently covered by Taiwan’s National Health Insurance Program. We excluded children who had received palivizumab prophylaxis for other indications, such as congenital heart disease and very preterm birth, enabling evaluation of natural RSV infection outcomes. Taiwan’s universal single-payer healthcare system and its associated research database facilitated the comprehensive identification of all patients with SMA and hospitalization episodes, significantly minimizing the risk of potential selection bias and incomplete data due to follow-up loss.

This study has several limitations. First, we relied solely on ICD codes to identify patients with SMA. Claims data cannot reliably differentiate SMA subtypes, lacks genetic information for accurate phenotyping, and does not capture the precise age of symptom onset. The burden of RSV in SMA may also be underestimated due to evolving disease classifications and historical high fatality rates in type 1 SMA. Our claims-based definition of mechanical ventilation captures any ventilation documented during the hospitalization episode but cannot distinguish between ventilation newly initiated for the acute infection and continuation of pre-existing home ventilatory support. This limitation is inherent to claims-based respiratory research, as prior large-scale studies have similarly relied on ICD codes for mechanical ventilation as a severity proxy without differentiating new initiation from baseline use [20, 27]. Given that patients with SMA, particularly those with earlier onset, may already require long-term ventilatory support, the reported ventilator use rates may partly reflect baseline respiratory status. Nevertheless, the markedly higher rates observed in patients with SMA compared to the general population (54.5% vs. 1.7%) suggest that RSV infection imposes a substantial additional respiratory burden even after considering potential baseline ventilator dependence. Second, our birth cohort follow-up period may not accurately reflect infection patterns among school-age children, and individual differences in pulmonary function, which influence infection risk and severity, cannot be determined from claims data [28]. Third, because only the final approximately 1 year of our 13-year study period overlapped with the DMT availability and the initial reimbursement criteria were highly restrictive, the impact of DMT on our cohort is likely minimal. Therefore, our findings primarily reflect the burden of RSV infection among patients with SMA in the pre-treatment era. Fourth, our study may underestimate the true burden of RSV infection in patients with SMA because viral testing practices are risk-stratified in Taiwan. Although all identified RSV hospitalizations occurred in patients under 18 years of age, this pattern may reflect differences in testing practices rather than true epidemiology, as testing in older children and adults is less systematic [29]. Furthermore, the common reliance on rapid antigen tests, which possess lower sensitivity compared to PCR, may result in false-negatives being recorded as “unspecified LRTI” in the NHIRD [30]. Lower viral loads and delayed clinical presentations in older patients further compound under-reporting, where the virus may no longer be detectable by the time of hospitalization [31]. Although we included LRTI with unspecified pathogens to account for potential under-detection, the real-world RSV burden, particularly in adult patients with SMA, may be higher than observed. These diagnostic limitations also apply to the pediatric population; therefore, the reported 9.8% RSV hospitalization rate in our birth cohort should be interpreted as a minimum estimate, with the true burden likely being higher. Further studies are needed to characterize the RSV disease burden in adult patients with SMA to provide more evidence-based practice guidelines.

## Conclusion

This nationwide study provides valuable insights into RSV infection patterns among patients with SMA, uncovering unique characteristics compared to the general population, such as higher hospitalization rates, increased ICU admissions, and distinct age-related severity trends. These findings underscore the significant respiratory vulnerability of patients with SMA, particularly within pediatric populations, and highlight the urgent need for targeted preventive strategies for children over two years old. While modern SMA treatments have substantially improved outcomes, our results emphasize the continued importance of RSV prevention, especially for older children who remain at elevated risk for severe complications. Future research into the efficacy of RSV monoclonal antibody therapies in children over two years old is crucial for optimizing patient outcomes and healthcare resource allocation, particularly during critical developmental stages and seasonal peaks.

## Supplementary Information

Below is the link to the electronic supplementary material.

Supplementary Material 1

## Data Availability

The data that support the findings of this study are available from the Health and Welfare Data Science Center (HWDC), Ministry of Health and Welfare, Taiwan. Restrictions apply to the availability of these data, which were used under license for the current study and are therefore not publicly available. Data are available from the authors upon reasonable request and with permission from the HWDC.
