# Supplementary material for: Burden of respiratory syncytial virus in spinal muscular atrophy: a retrospective nationwide cross-sectional and birth cohort study
Source: Orphanet J Rare Dis. 2026 Jul 14;21:351. doi: 10.1186/s13023-026-04494-0 (PMC13647703; doi:10.1186/s13023-026-04494-0)
Supplement: Supplementary file 1 [file 13023_2026_4494_MOESM1_ESM.docx]

| Variables | ICD-9-CM | ICD-10-CM / ICD-10-PCS |
| --- | --- | --- |
| SMA | 335.10, 335.11, 335.19 | G120, G121, G129 |
| Comorbidities |  |  |
| Hypertension | 401-405 | I10-I15 |
| Diabetes | 250 | E08-E13 |
| Hyperlipidemia | 272.0x-272.4x, 272.9x | E78.0x-E78.5x |
| Mechanical ventilation | V46.1, 93.90 | Z99.1, 5A09557 |
| Spinal fusion surgery | 810.5, 810.8 | 0RG80J1, 0RGA0J1, 0SG10J1, 0SG30J1 |
| Neonatal condition (within 7 days after birth) | | |
| TTNB | 770.6 | P22.1 |
| Jaundice | 99.83, 99.01 | 6A600ZZ, 6A601ZZ, 30233H1, 30243H1, 30253H1, 30263H1 ,302A3H1 |
| Outcome | | |
| RSV | 466.11, 480.1, 079.6 | J205, J210, J121, B974 |
| LRTI | 46619, 480.0, 480.2, 480.8-480.9, 4870 | J219, J218, J211, J129, J120, J128.9, J122, J123, J100.0, J100.1, J100.8, J110.0, J110.8 |

**E-table 1** Definitions of study variables

**E-table 2. Characteristics for Cross-Section SMA Patients.**

|  | SMA (n=914) |
| --- | --- |
| Characteristics |  |
| Hypertension | 176 (19.3) |
| Diabetes | 107 (11.7) |
| Hyperlipidemia | 169 (18.5) |
| Mechanical ventilation | 183 (20.0) |
| Spinal fusion surgery | 54 ( 5.9 ) |

**E-figure 1. Flowchart of the study population in cross section study and birth cohort.**
